# Local and Remote Chemogenetic Suppression of Hippocampal Seizures in Rats

**DOI:** 10.2174/1570159X22999240131122455

**Published:** 2024-02-07

**Authors:** Donghong Li, Xi Yan, Yue Xing, Jiaqing Yan, Junling Wang, Herui Zhang, Jiaoyang Wang, Xiaonan Li, Zhumin Su, Horace Hao Loh, Xiaofeng Yang, Xiaohong Chen

**Affiliations:** 1 Department of Neurology, Third Affiliated Hospital, Sun Yat-sen University, Guangzhou, Guangdong, China;; 2 Department of Neurology, Henan Provincial People’s Hospital, Zhengzhou University People’s Hospital, Henan University People’s Hospital, Zhengzhou, Henan, China;; 3 Guangzhou Laboratory, Guangzhou, Guangdong, China;; 4 College of Electrical and Control Engineering, North China University of Technology, Beijing, China

**Keywords:** Chemogenetics, epileptic network, clozapine-N-oxide, clozapine, anterior nucleus, hippocampal seizures

## Abstract

**Background:**

Innovative treatments of refractory epilepsy are widely desired, for which chemogenetic technology can provide region- and cell-type-specific modulation with relative non-invasiveness.

**Objectives:**

We aimed to explore the specific applications of chemogenetics for locally and remotely networks controlling hippocampal seizures.

**Methods:**

A virus coding for a modified human Gi-coupled M4 muscarinic receptor (hM4Di) on pyramidal cells was injected into either the right hippocampal CA3 or the bilateral anterior nucleus of the thalamus (ANT) in rats. After one month, seizures were induced by 4-aminopyridine (4-AP) injection into the right CA3. Simultaneously, clozapine-N-oxide (CNO) (2.5 mg/kg) or clozapine (0.1 mg/kg), the specific ligands acting on hM4Di, were injected intraperitoneally. We also set up hM4Di control and clozapine control groups to eliminate the influence of viral transfection and the ligand alone on the experimental results.

**Results:**

For both local and remote controls, the mean seizure duration was significantly reduced upon ligand application in the experimental groups. Seizure frequency, on the other hand, only showed a significant decrease in local control, with a lower frequency in the clozapine group than in the CNO group. Both the effects of CNO and clozapine were time-dependent, and clozapine was faster than CNO in local seizure control.

**Conclusion:**

This study shows the potency of chemogenetics to attenuate hippocampal seizures locally or remotely by activating the transfected hM4Di receptor with CNO or clozapine. ANT is suggested as a potentially safe chemogenetic application target in the epileptic network for focal hippocampal seizures.

## 1 INTRODUCTION

New therapeutic approaches to refractory epilepsy are needed [1-3]. Although the mechanism underlying epilepsy remains unknown, the emergence of seizures has been widely attributed to an excitatory shift out of the neuronal activity balance as validated by several gene therapies utilizing preclinical models [4]. Several gene therapies that alter the balance between neuronal excitation and inhibition have been validated using preclinical models [5-8]. Among them,a recently developed chemogenetic technology, Designer Receptors Exclusively Activated by Designer Drugs (DREADDs), not only allows region- and cell-type specific inhibition and excitation of neuronal activities but also has the advantage of being relatively non-invasive [9, 10]. A selective promoter system carried by an injected recombinant adeno-associated virus (rAAV) vector achieves the expression of DREADDs. The most commonly used DREADDs are modified human muscarinic receptors engineered to be insensitive to the native ligand acetylcholine but activated by the designer drug clozapine‐N‐oxide (CNO). Depending on the type of receptors, neurons are activated (hM3Dq) or inhibited (hM4Di) [11-13].

Following the pioneering study of Katzel *et al.* [14] on controlling epileptic seizures through chemogenetic inhibiting pyramidal neurons in the seizure-onset zone of the motor cortex, numerous studies for epilepsy treatment [15-17] have been investigated in animal studies. Most chemogenetic studies have used CNO, the inactive metabolite of the atypical antipsychotic drug clozapine, as the ligand. However, Gomez *et al.* [18] reported that CNO can be converted into clozapine, which can quickly pass through the blood-brain barrier and subsequently act as a ligand to activate DREADDs in animal models. In addition, CNO has also been suggested to exert clozapine-like behavioral effects in both mice and rats [19]. As an activator of hM4Di, clozapine is effective in many patients with resistant schizophrenia [20]. Unlike CNO, clozapine has been approved by the U.S. Food and Drug Administration for clinical use. Desloovere *et al.* [21] have attempted to evaluate the effect of clozapine on chemogenetic seizure suppression by expressing DREADDs universally among the excitatory pyramidal and granule cells in the mouse sclerotic hippocampus and repeated clozapine exposure led to almost complete seizure suppression.

Epilepsy has been increasingly recognized to reflect abnormalities in neuronal networks, or the so-called epileptogenic network, characterized by seizure-generating “foci” embedded in a web of structural and functional connections [22-24]. One region that has attracted particular attention is the anterior nucleus of the thalamus (ANT), which is closely connected to the mesial temporal structures *via* the fornix, mammillothalamic tracts, and thalamocortical radiations, making it a tempting target for the modulation of overall thalamocortical excitability and the limbic seizure network [25-27]. Both localized electroencephalogram (EEG) activity recorded in the temporal region [28] and evoked potentials recorded with depth electrodes in the hippocampus in response to ANT stimulation [29, 30] have confirmed a strong connection among these structures. Previous studies have confirmed that electrical stimulation of the ANT can effectively control epileptic seizures [31-34]. However, electrical stimulation cannot elucidate the specific neuron and mechanism that takes effect during regulation. In addition, few studies have directly compared the effect of local control based on hippocampal circuits and those of remote control at ANT based on limbic circuits in the same hippocampal seizure model.

Here, we selectively targeted pyramidal cells in the right hippocampal CA3 (seizure onset zone for local control) or bilateral ANT (remote control) to express hM4Di receptors using Ca^2+^/calmodulin-dependent kinase II alpha (CaMKIIα). We sought to evaluate the efficacy of intraperitoneal administration of CNO or clozapine in attenuating hippocampal seizures induced by 4-aminopyridine (4-AP) injection into the CA3 in rats. We also examined the time effects of CNO and clozapine on electrographic seizures and potential cognitive impairments. Our study will underscore chemogenetics as a novel approach for focal epilepsy by inhibiting the seizure focus or nodes in the occult epileptic network, which may open new avenues for the precise treatment of refractory epilepsy.

## 2 MATERIALS AND METHODS

### 2.1 Animals and Chemicals

Experiments were performed on a total of eighty adult male Sprague-Dawley rats (250-350g) at specific pathogen-free levels. The experimental protocols were approved by the ethical guidelines of the Sun Yat-Sen University and Guangzhou Laboratory Animal Experimentation Committee. Viruses were purchased from HanBio Co. (Shanghai, China). CNO was purchased from Abcam Biochemicals (Cambridge, UK), and clozapine was purchased from Selleck Chemicals (Houston, TX, USA). All other chemicals were purchased from Sigma-Aldrich Co. (St Louis, MO, USA).

### 2.2 Experimental Groups

The experiment was divided into two parts: local and remote seizure control. The rats in each part were then divided into five groups, including two treatment groups (CNO and clozapine) and three control groups (model, hM4Di, and clozapine control) to alleviate the influence of viruses and ligands on the experimental results.

### 2.3 Stereotaxic Virus/Vehicle Injections

Rats were anesthetized with isoflurane (2%) and placed on a Stoelting stereotaxic frame (Stoelting, Wood Dale, IL, USA). All coordinates were measured from the bregma according to the atlas of Paxinos and Watson [35]. For local seizure control, a recombinant adeno-associated virus (rAAV2-CaMKIIα-hM4Di-mcherry, 1×10*12 infectious particles per ml, 1.5 μL) was injected into the right hippocampal CA3 area (AP -3.8, ML -4.0, DV -4.0) by a Nanoject II injector (Drummond Scientific, Broomall, PA, USA) with a matched glass micropipette (tip diameter 100 µm). For remote seizure control, the same amount of virus was injected bilaterally into the ANT (AP -2.0, ML ±1.5, DV -5.5, 1.5 μL each side) through a cannula guide (inner diameter 0.25 mm and outer diameter 0.41 mm; RWD Life Science, Shenzhen, China) connected *via* tubing to a Hamilton syringe (Hamilton, Bonaduz, Switzerland). Rats in the clozapine control group were injected with 1.5 μL saline at the same site. The injection was carried out over 15 min at a rate of 0.1 μL/min to minimize tissue damage and maintained for another 5 min to minimize leakage and allow for viral diffusion.

### 2.4 Learning and Memory Tests

To determine whether virus vector injection of hippocampal epileptic focus or bilateral ANT could induce cognitive impairments, we examined the memory function of experimental rats. One month after the virus injection, the Morris water maze test was performed on the virus-transfected and normal rats with matching weights to examine learning and memory function. The Morris water maze test was performed in a cylindrical tank (150 cm × 90 cm) filled with opaque water obscuring a platform (water 2 cm above platform height) located in the center of a quadrant. A video tracking system (Coulbourn Instruments, USA) was used to trace movements in the calculations of latency and distance traveled. During the five consecutive trial days, rats had four acquisition learning trials per day (60 s each) to find the hidden platform. If the platform was not found within this time period, rats were guided to the platform and left on the platform for 15 s. On the sixth day, the platform was removed to assess memory retention, and rats were given a single 60 s extinction trial [36].

### 2.5 Hippocampal Seizure Model and CNO or Clozapine Administration

**Fig. (1):**
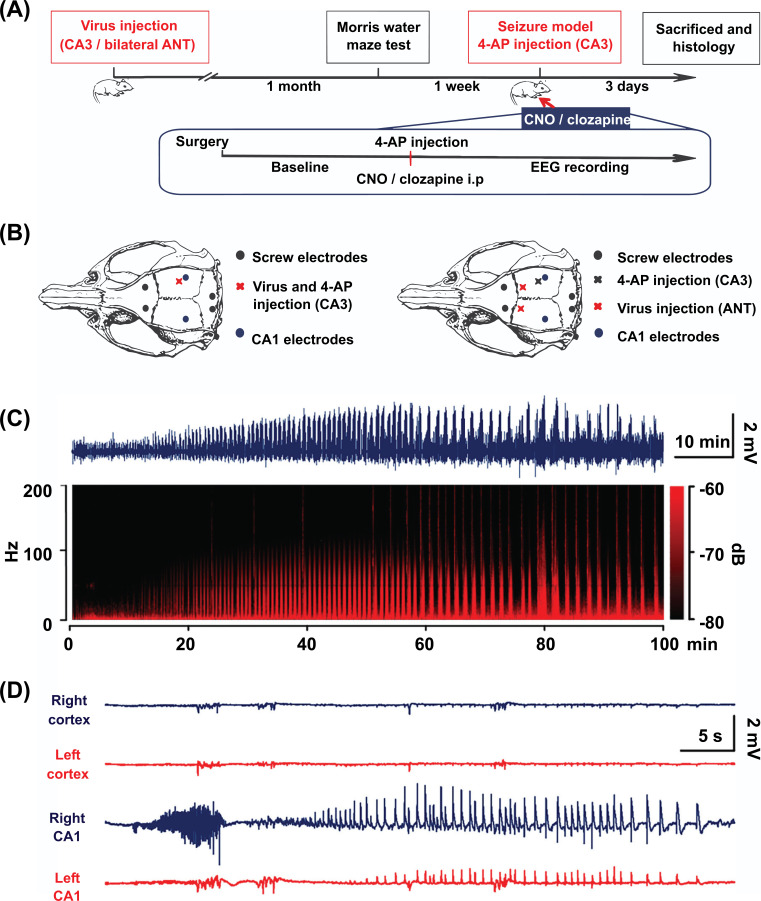
Schematic diagram of experimental schedule and 4-AP induced hippocampal seizure model. (**A**) Experimental scheme. (**B**) Schematic diagram of the virus injection site, the placement of electrodes, and the 4-AP injection site. A schematic diagram for local control (left) showed the virus, and 4-AP was injected into the same site (right hippocampal CA3). As for remote control, the virus was injected into bilateral ANT, while 4-AP was injected into the right hippocampal CA3. (**C**) Representative EEG data within 100 min after 4-AP administration (top) and the corresponding spectrum (bottom) of right hippocampal CA1 in the model group. (**D**) An example of 4-AP induced hippocampal seizures. CNO, clozapine-N-oxide; EEG, electroencephalogram; 4-AP, 4-aminopyridine; ANT, anterior nucleus of the thalamus.

Rats were anesthetized with isoflurane and mounted in the stereotaxic apparatus. Two stainless steel screw electrodes and two hand-made tungsten wire electrodes (diameter 177 μm, HFV-Natural, CFW, USA) were symmetrically implanted into the skull over bilateral motor cortex areas and bilateral hippocampal CA1 (AP -5.2, ML ± 4.5, DV -3.4), respectively. Two stainless steel screw electrodes were implanted into the occiput area, with the neck muscles serving as a reference and ground (Fig. **1**). The EEG signals were recorded and digitized using a PowerLab 8/35 system (AD instrument, Springs, Colorado, USA) with a sampling rate of 2 kHz and high and low pass-filtering set at 1 Hz and 500 Hz, respectively. After a 30-minute baseline EEG recording, 0.5 µL 4-AP (25 mM in saline) was injected into the right hippocampal CA3 area (AP -3.8, ML -4.0, DV -4.0) using the Nanoject II injector to induce focal hippocampal seizures. The injection was carried out over a 5-minute period at a speed of 0.1 μL/min to minimize tissue damage, and the pipette was maintained for an additional 5 min to minimize leakage of the 4-AP. At the same time as the 4-AP injection, CNO (2.5 mg/kg, diluted at 1 mg/ml in 1% DMSO/saline vehicle) or clozapine (0.1 mg/kg, diluted at 0.1 mg/ml in 1% DMSO/saline vehicle) was intraperitoneally injected into the rats in the CNO or clozapine and clozapine control groups. EEG was continuously recorded for two consecutive hours. All experimental data were stored on a personal computer and analyzed offline using the LabChart 8 software and MATLAB (Math Works, Natick, MA, USA).

### 2.6 EEG Analysis

The classification of epileptic seizures was determined by two reviewers (Y.X. and J.W.) blinded to the group assignment of rats. The efficacy of CNO and clozapine in controlling seizures was analyzed and compared between the groups. To explore the pharmacodynamic potency of CNO and clozapine, the EEG was divided into consecutive 10-minute periods for analysis after 4-AP injection. Additionally, to explore the effects of chemogenetics on EEG power in the epileptogenic zone during seizures, a fast Fourier transformation was used to calculate the power spectral density (PSD) of seven different frequency bands during seizures: delta (1-4 Hz), theta (4-8 Hz), alpha (8-13 Hz), beta (13-30 Hz), gamma (30-80 Hz), ripples (80-200 Hz), and fast ripples (200-500 Hz). Due to large individual variability, we normalized the PSD value. Using three non-seizure EEG segments prior to the injection of 4-AP as the baseline.

Seizures in patients with hippocampal seizures are mainly characterized by two distinct electrographic onset patterns: low-voltage, fast (LVF) and hypersynchronous (HYP) [37], characterized respectively as a sentinel spike followed by low-amplitude, high-frequency activity or preictal spiking at a frequency of approximately 2 Hz [38]. We calculated their percentage in different treatment groups to explore the effects of chemogenetics on different neuronal activities.

### 2.7 Fluorescence and Immunohistochemical Analysis

Three days after the seizures were induced, rats were euthanized with an overdose of isoflurane and then perfused transcardially with saline and 4% paraformaldehyde. The brains were immediately removed and post-fixed in 4% phosphate-buffered paraformaldehyde at 4°C overnight. Afterward, the brains were equilibrated in a 30% (w/v) sucrose solution. Frozen coronal 30 μm brain sections were prepared using a cryostat microtome (Leica, Germany). mCherry fluorescence was used to determine the accuracy of virus injection. Then all slices were permeabilized in phosphate buffer saline (PBS) and 0.3% Triton X-100 for 30 min, blocked with 10% normal goat serum for 1 h on a shaker and incubated for 2 days with primary antibodies against CaMKIIα (rabbit, 1:500) to confirm the type of transfected neurons. Following three further washes in PBS, the sections were incubated in secondary antibodies (1:500, Alexa-488) overnight and washed in PBS again (3 times). Images were obtained using a confocal microscope. We used Nissl staining, TdT-mediated dUTP-biotin nick-end labeling (TUNEL) staining, and glial fibrillary acidic protein (GFAP) staining to detect necrotic injury, apoptotic death, and glial proliferation, respectively.

### 2.8 Statistical and Data Analyses

All experimental data were analyzed using SPSS software 22.0 (IBM, Armonk, New York, USA) and are presented as mean ± standard error of the mean (SEM). In the Morris water maze experiment, repeated measures analysis of variance was used to compare the differences in escape latency and distance between the virus and control groups on the 5 training days. We used two independent sample t-tests to analyze the differences in the frequency of platform crossing and the percentage of time spent in the target zone on the sixth day between the virus and control groups. We used one-way, two-tailed ANOVA followed by the least significant difference post hoc test to compare the effects of CNO and clozapine on the control of focal hippocampal seizures among the groups. Statistical significance was set at *p* < 0.05.

## 3 RESULTS

### 3.1 Virus Transfection and DREADD Expression

**Fig. (2):**
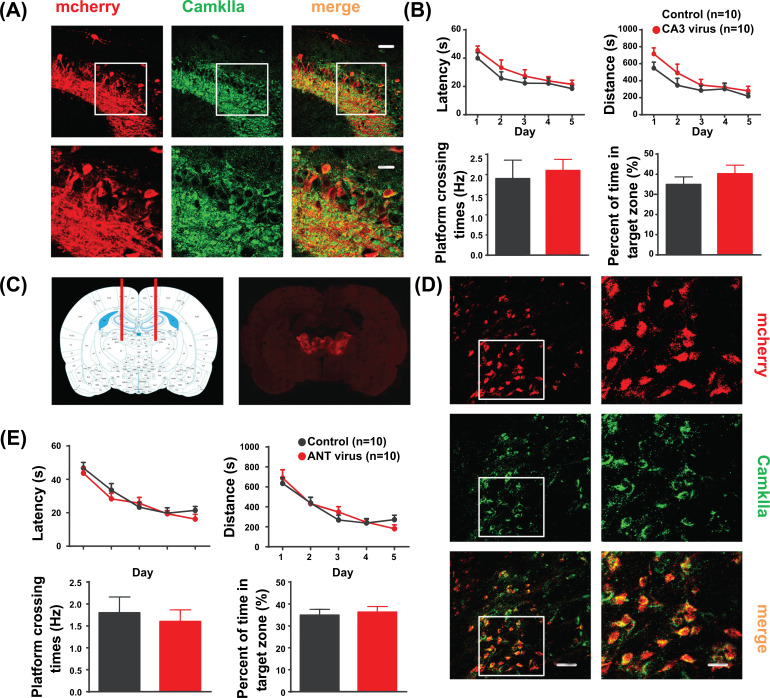
Virus transfection and the Morris water maze test. (**A**) Expression of hM4Di-cherry in right CA3, visualized with mcherry (red), anti-CamKIIα (green), and colocalization of virus transfection and glutamatergic neurons (merge). Scale, above 50 μm, below 25 μm. (**B**) The Morris water maze test results show that there was no statistical difference between control rats and hippocampus virus-transfected rats. (**C**) Virus injection site in bilateral ANT visualized with the whole brain slice of rat brain atlas (left) and image splicing (right) of photomicrographs within virus infection of bilateral ANT. (**D**) Representative photomicrographs showing mCherry (red) fluorescence, anti-CamKIIα (green), and colocalization of virus transfection and glutamatergic neurons (merge) within the bilateral ANT. Scale, left 50 μm, right 25 μm. (**E**) The Morris water maze test results show there was no statistical difference between control rats and ANT virus-transfected rats. ANT, anterior nucleus of the thalamus; CaMKIIα, Ca^2+^/calmodulin-dependent kinase II alpha; hM4Di, human Gi-coupled M4 muscarinic receptor.

According to the distribution results of mCherry fluorescence in the brain section, seven rats were excluded because of the inaccurate injection site of the virus. Eventually, eighteen (85.71%) hippocampal virus-transfected rats and eighteen (81.82%) ANT virus-transfected rats were used for statistical analysis, evenly divided into hM4Di, CNO, and clozapine groups. Representative photomicrographs showed the expression of hM4Di-mCherry on pyramidal neurons of the right hippocampal CA3 and bilateral ANT, which were visualized with mCherry, anti-CamKIIα, and colocalization of virus transfection and glutamatergic neurons (Fig. **2**).

### 3.2 Virus Vector Injection did not change Learning and Memory Abilities

The results of the Morris water maze test showed that the escape latency and distance to reach the hidden platform decreased over the five training days at a similar rate in both hippocampus virus-transfected rats and control rats (*p >* 0.05, Fig. **2**). In addition, there was no difference in the frequency of platform crossing between control rats (1.9 ± 0.5) and hippocampus virus-transfected rats (2.1 ± 0.3, *p >* 0.05), or in the percentage of time spent in the target zone between control rats (34.9 ± 3.8%) and hippocampus virus-transfected rats (40.2 ± 4.3%, *p >* 0.05, Fig. **2**). Similarly, there was no significant difference in escape latency, distance during five training days, frequencies of platform crossing on the sixth day, and the percentage of time spent in the target zone between the control group and the ANT virus-transfected group (*p >* 0.05, Fig. **2**).

### 3.3 4-AP-induced Focal Hippocampal Seizures in Rats

A seizure was defined as an abrupt change in EEG frequency and amplitude. Distinct seizure events were defined by 10-second intervals of event separation. The interictal period was defined as the interval duration between two consecutive seizures. The classification of epileptic seizures was determined by two reviewers (X.Y. and J.W.) in a blind manner (without knowledge of the group assignment of the rats).

We did not detect any seizures before the 4-AP injection. Within 10.3 ± 3.0 minutes of 4-AP injection, the ipsilateral electrode in the right CA1 began to detect stable recurrent electrographic focal seizures, which lasted up to 2.5 hours. The majority of rats were injected with 4-AP experienced abnormal seizures in about 80 minutes post-administration (Figs. **1C**, **D**). We, therefore, only considered data within 70 minutes of 4-AP injection for analysis. The seizure frequency was 63.2 ± 4.1 within the whole 70 minutes, the mean seizure duration (MSD) was 28.8 ± 6.64 s, and the mean interictal duration (MID) was 29.8 ± 3.7 s.

### 3.4 hM4Di Expression or Clozapine Administration only did not Alter Seizures

We compared the latency of hippocampal seizures, MSD, seizure frequency (SF), and total seizure duration (TSD) between the hM4Di and clozapine control and model groups. The results showed no statistically significant differences in any parameter.

### 3.5 Local Control of Focal Hippocampal Seizures using Chemogenetic Technique

**Fig. (3):**
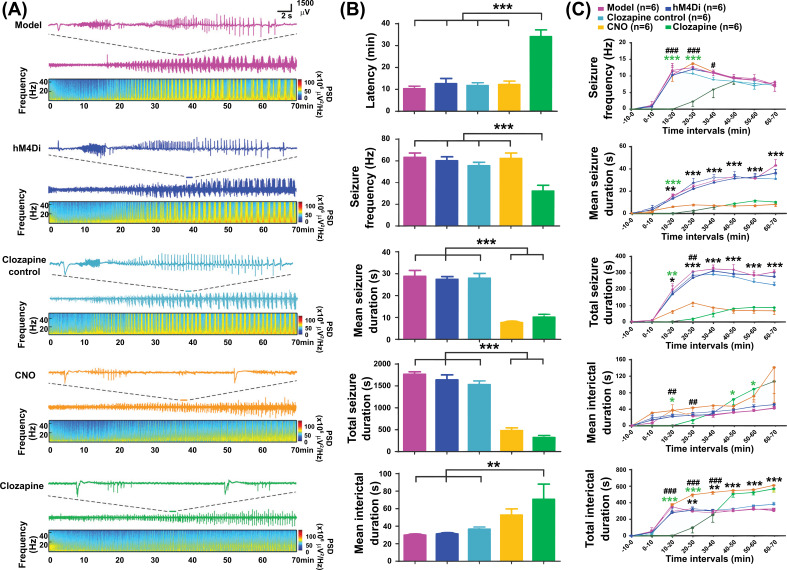
Chemogenetic local control of focal hippocampal seizures. (**A**) EEG recording and analysis results of each group are shown. The first row in each group is a 4-AP-induced typical hippocampal seizure. The second row shows EEG recordings from 0-70 min after 4-AP injection. The third row is the result of spectrum analysis corresponding to the second row of EEG recording. (**B**) Effects of chemogenetic inactivation of pyramidal neurons in right hippocampal CA3 for 4-AP induced focal hippocampal seizures on latency, seizure frequency, mean seizure duration, total seizure duration, and mean interictal duration of seizures within 70 min. (**C**) Temporal evolution of seizure frequency, mean seizure duration, total seizure duration, mean interictal duration and total interictal duration from top to bottom, in model, hM4Di, clozapine control, CNO and clozapine group trials (consecutive 10-min intervals before and after 4-AP and CNO or clozapine injection. ***represents the difference between the two treatment groups and the control groups, *p* < 0.001, **represents the difference between CNO and three control groups, *p* < 0.01, *p < 0.05, ***represents the difference between clozapine and three control groups, *p* < 0.001, *p < 0.05, ^###^represents the difference between CNO and clozapine group, *p* < 0.001, ^##^*p* < 0.01, ^#^*p* < 0.05. CNO, clozapine‐N‐oxide; hM4Di, human Gi-coupled M4 muscarinic receptor.

In the local seizure control part, the MSD was significantly reduced in both the CNO and clozapine groups compared with that in the model, hM4Di, and clozapine control groups (from 28.8 ± 2.7 s, 27.4 ± 1.3 s, and 27.9 ± 2.2 s to 7.6 ± 0.8 s and 10.2 ± 1.3 s, *p* < 0.001, Fig. **3**). However, the latency of seizures was significantly prolonged in the clozapine group compared to the three control groups (from 10.3 ± 1.2 min, 12.6 ± 2.4 min, and 11.7 ± 1.4 min to 34.1 ± 3.1 min, *p* < 0.001, Fig. **3**), but not in the CNO group (12.2 ± 1.6 min). Within 70 min after 4-AP injection, SF was significantly lower in the clozapine group than in the model, hM4Di, and clozapine control groups (from 63.2 ± 4.1, 60.0 ± 3.8, and 55.5 ± 3.2 to 32.2 ± 5.3, *p* < 0.001, Fig. **3**) but not in the CNO group (62.2 ± 5.1). In contrast, the clozapine group had a significantly prolonged MID compared with the three control groups (from 29.8 ± 1.5 s, 31.1 ± 1.6 s, and 36.6 ± 2.6 s to 70.7 ± 17.3 s, *p* < 0.01, Fig. **3**). While the prolonged MID in the CNO group did not reach statistical significance, a clear trend was observed.

When we divided the EEG into consecutive 10 min segments, we found a very sharp time-response function for CNO and clozapine in this experiment. The results showed that seizure activity was, on average, stronger in the model, hM4Di, and clozapine control groups than in the CNO and clozapine groups at 10-minute intervals. While CNO took effect 10-20 minutes post injection as opposed to the immediate reaction of clozapine, both drugs reached an equivalent level of effectivity by 40-50 minutes post injection (Fig. **3**). The first 10 min after CNO injection showed no significant difference in terms of MSD and SF compared to those of the model, hM4Di, and clozapine control groups (*p >* 0.05). Both the MSD and TSD in the 10-70 min intervals decreased in the CNO group compared to those of the three control groups (*p* < 0.05), and the TID was significantly prolonged in the 20-70 min intervals (*p* < 0.05). In the clozapine group, the SF significantly decreased within 40 min (*p* < 0.05) after administration, coupled with prolonged latency, suggesting an immediate effect of clozapine injection.

### 3.6 Remote Control of Focal Hippocampal Seizures using Chemogenetic Technique

**Fig. (4):**
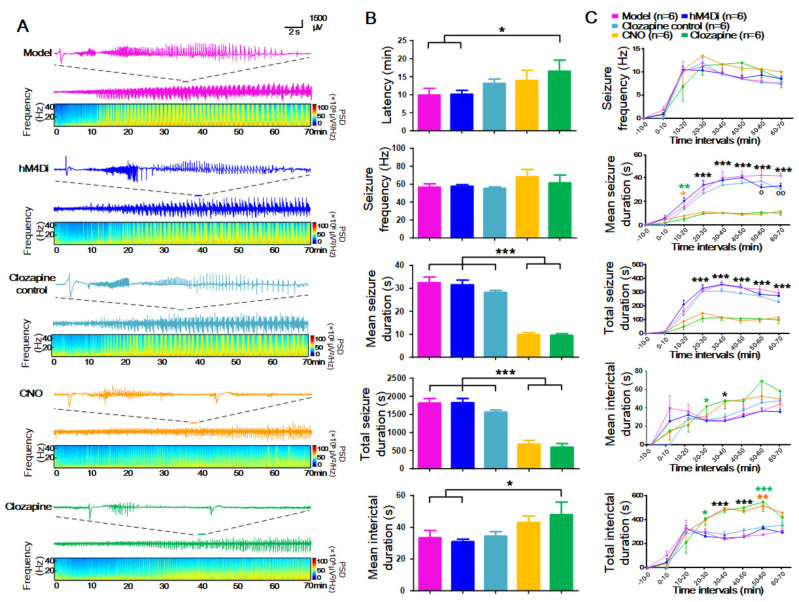
Chemogenetic remote control of focal hippocampal seizures. (**A**) EEG recording and analysis results of each group are shown. The first row in each group is a 4-AP-induced typical hippocampal seizure. The second row shows EEG recordings from 0-70 min after 4-AP injection. The third row is the result of spectrum analysis corresponding to the second row of EEG recording. (**B**) Effects of chemogenetic inactivation of pyramidal neurons in bilateral ANT for 4-AP induced focal hippocampal seizures on latency, seizure frequency, mean seizure duration, total seizure duration and mean interictal duration of seizures within 70 min. (**C**) Temporal evolution of seizure frequency, mean seizure duration, total seizure duration, mean interictal duration and total interictal duration from top to bottom, in model, hM4Di, clozapine control, CNO and clozapine group trials (consecutive 10-min intervals before and after 4-AP and CNO/clozapine injection. ***represents the difference between the two treatment groups and three control groups, *p* < 0.001, *p < 0.05, **represents the difference between CNO and three control groups, *p* < 0.01, *p<0.05, *** represents the difference between clozapine and three control groups, P < 0.001, **p < 0.01, *p < 0.05, oo represents the difference between model and hM4Di group, *p* < 0.01, o *p* < 0.05. CNO, clozapine‐N‐oxide; ANT, anterior nucleus of the thalamus; hM4Di, human Gi-coupled M4 muscarinic receptor; EEG, electroencephalogram.

In the remote seizure control part, compared to the model, hM4Di, and clozapine control groups, the MSD was significantly reduced in the CNO and clozapine groups (from 32.5 ± 2.5 s, 31.6 ± 2.1 s, and 28.3 ± 0.9 s to 9.8 ± 0.8 s and 9.6 ± 0.7 s. *p* < 0.001, Fig. **4**). The latency of seizures was significantly prolonged in the clozapine group compared with the model and hM4Di groups (from 9.9 ± 1.9 min and 10.1 ± 1.1 min to 16.5 ± 3.1 min, *p* < 0.05). However, there was no significant difference in SF among the five groups within 70 min. While the MID of CNO-administrated rats did not show statistical significance compared to the controls, clozapine administration significantly prolonged the MID compared to the model and hM4Di groups (from 33.4 ± 4.5 s and 30.9 ± 1.6 s to 48.0 ± 7.9 s, *p* < 0.05, Fig. **4**).

After dividing the EEG into consecutive 10-minute segments, no statistical significance was found in terms of SF, MSD, or MID among the groups within the first 10 min interval. Both CNO and clozapine took effect 10-20 minutes post injection and had an almost equivalent effect. During the 10-20 min intervals, compared to the model, hM4Di, and clozapine control groups, MSD significantly decreased in the CNO and clozapine groups (*p* < 0.05). The MSD and TSD in the 20-70 min intervals of the CNO and clozapine groups clearly decreased (*p* < 0.001) compared with the control groups. The TID was significantly prolonged in the 30-70 min intervals in the CNO group (*p* < 0.05) and only in the 30-60 min intervals in the clozapine group (*p* < 0.05, Fig. **4**).

### 3.7 Comparison between Local and Remote Control of Seizures using Chemogenetics

**Fig. (5):**
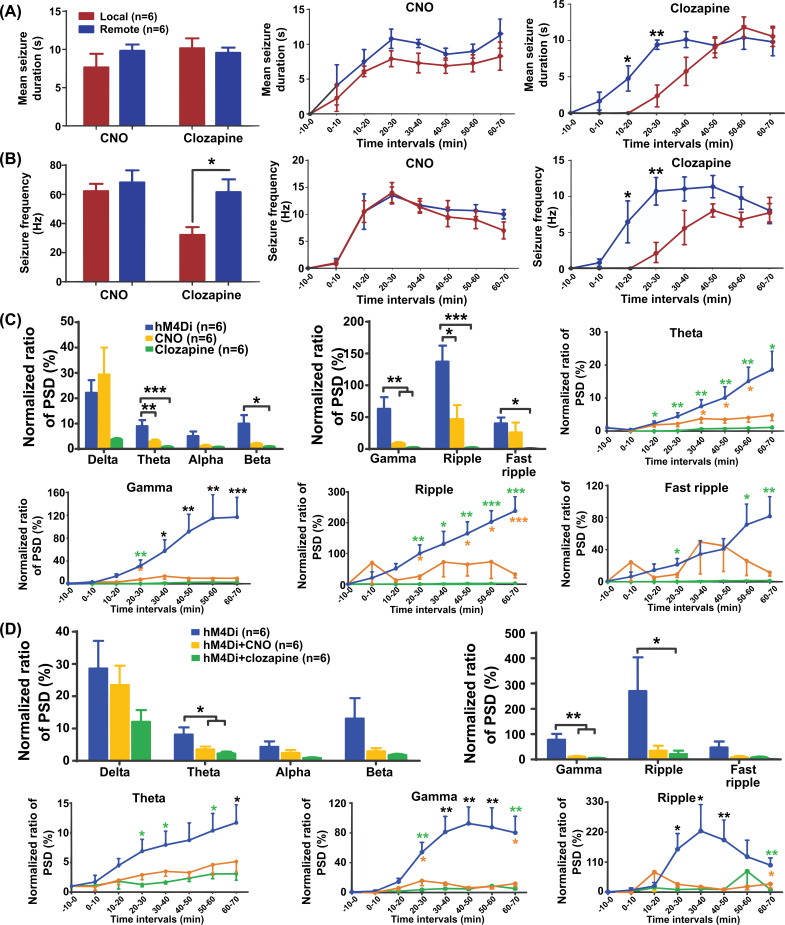
Comparison of chemogenetics effects in local and remote seizure control, and effects of chemogenetics on EEG power in different frequency bands in hippocampal seizures. (**A**) The effects of chemogenetics in local and remote control on mean seizure duration with either CNO or clozapine as ligands. (**B**) The effects of chemogenetics in local and remote control on seizure frequency with either CNO or clozapine as ligands. (**C**) Local effects of chemogenetics on EEG power in different frequency bands in hippocampal seizures within 70 min after 4-AP injection, and temporal evolution of the effects of chemogenetics on power spectrum of seizures by consecutive 10-min-intervals. (**D**) Remote effects of chemogenetics on EEG power in different frequency bands in hippocampal seizures within 70 min after 4-AP injection, and temporal evolution of the effects of chemogenetics on the power spectrum of seizures by consecutive 10-min intervals. ***represents the difference between the two treatment groups and hM4Di group, *p* < 0.001, **p < 0.01, * *p* < 0.05; ***represents the difference between CNO and hM4Di group, *p* < 0.001, *p < 0.05; ***represents the difference between clozapine and hM4Di group, *p* < 0.001, **p < 0.01; *p < 0.05. CNO, clozapine‐N‐oxide; hM4Di, human Gi-coupled M4 muscarinic receptor; EEG, electroencephalogram; PSD, power spectral density.

We compared the effects of local and remote control of focal hippocampal seizures using chemogenetic techniques. First, the result of the 70-minute recording period showed no significant differences in MSD and SF of all control groups between the two seizure control parts. When we compared the efficacy of chemogenetic local and remote seizure control with either CNO or clozapine as ligands, we found that SF was significantly lower in local seizure control than in remote seizure control upon administration of clozapine (*p* < 0.05, Fig. **5**). However, no significant differences were found in SF between local and remote seizure control when CNO was administered (*p >* 0.05, Fig. **5**). In addition, MSD was not significantly different under either local or remote seizure control, regardless of whether CNO or clozapine was administered (*p >* 0.05, Fig. **5**).

In addition, we further compared the effects between local and remote control in each 10 min interval in the CNO and clozapine treatment groups. The results showed no statistically significant difference in SF or MSD between the local and remote seizure control parts at each 10 min interval when CNO was administered (*p >* 0.05, Fig. **5**). However, compared with the remote seizure control part, reductions in both SF and MSD in the local seizure control part were statistically significant at 10-30 min intervals when clozapine was administered (*p* < 0.05, Fig. **5**).

### 3.8 Chemogenetic Suppression on the Power Spectrum of EEG

In the local control of hippocampal seizures, both CNO and clozapine administration decreased the ratio of PSD in the theta, gamma, and ripple bands compared to the hM4Di group (*p* < 0.05, Fig. **5**). In particular, the ratio of PSD in the theta band was statistically reduced during 30-60 minutes in the CNO group (*p* < 0.05), while such a decrease took place at 10-70 minutes after clozapine administration (*p* < 0.05). The ratio of PSD in the gamma band was reduced during the 20-70 min intervals in both the CNO and clozapine groups (*p* < 0.05). In the ripple band, such a decrease occurred at 20-30 and 40-70-minute intervals in the CNO group (*p* < 0.05) and in the 20-70-minute intervals in the clozapine group (*p* < 0.05) (Fig. **5**).

In the remote control of hippocampal seizures, the ratio of PSD in the theta and gamma bands decreased in both the CNO and clozapine groups compared to the hM4Di group (*p* < 0.05). Specifically, the ratio of PSD in the theta band was reduced during the 60-70-minute intervals in the CNO group (*p* < 0.05) and during the 20-40 and 50-70-minute intervals in the clozapine group (*p* < 0.05). The ratio of PSD in the gamma band was significantly reduced during the 20-70-minute intervals in both the CNO and clozapine groups (*p* < 0.05, Fig. **5**).

### 3.9 Effects of Chemogenetics on Onset Type of Seizures

**Fig. (6):**
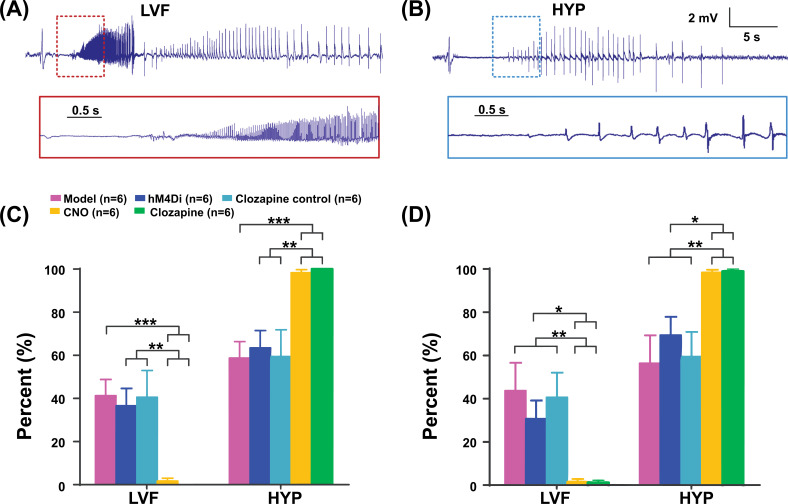
Effects of chemogenetics on onset type of seizures. (**A**) Representative examples of a LVF and (**B**) HYP seizure from the CA1 region of the right hippocampus in the model group. (**C**) Histogram showing LVF in the two treatment groups was reduced compared with that in the three control groups in local seizure control. On the contrary, HYP in the two treatment groups was increased compared with that in the three control groups. (**D**) Histogram showing LVF in the two treatment groups was reduced compared with that in the three control groups in remote seizure control, and HYP in the two treatment groups was increased compared with that in the three control groups. ***p < 0.001, **p < 0.01, *p < 0.05. LVF, low-voltage fast; HYP, hypersynchronous.

In the local control of focal hippocampal seizures, the LVF seizure-onset pattern was reduced in the two treatment groups compared to that in the model, hM4Di, and clozapine control groups (from 41.2 ± 7.6%, 36.6 ± 8.1%, and 40.6 ± 12.4% to 1.6 ± 1.4% and 0, *p* < 0.001, Fig. **6**). On the contrary, the HYP seizure-onset pattern in the two treatment groups was higher than in the three control groups (from 58.8 ± 7.6%, 63.4 ± 8.1%, and 59.4 ± 12.4% to 98.4 ± 1.4% and 100%, *p* < 0.001, Fig. **6**). In the remote control of focal hippocampal seizures, compared with that in the three control groups, the LVF onset in the two treatment groups reduced significantly (from 43.6 ± 13.0%, 30.6 ± 8.5%, and 40.6 ± 11.4% to 1.6 ± 1.2% and 1.1 ± 1.1%, *p* < 0.05, Fig. **6**). The HYP onset in the two treatment groups increased (from 56.4 ± 13.0%, 69.4 ± 8.5%, and 59.4 ± 11.4% to 98.4 ± 1.2% and 98.9 ± 1.1%, *p* < 0.05, Fig. **6**).

### 3.10 Applied Chemogenetics Showed no Neuropathological Changes

**Fig. (7):**
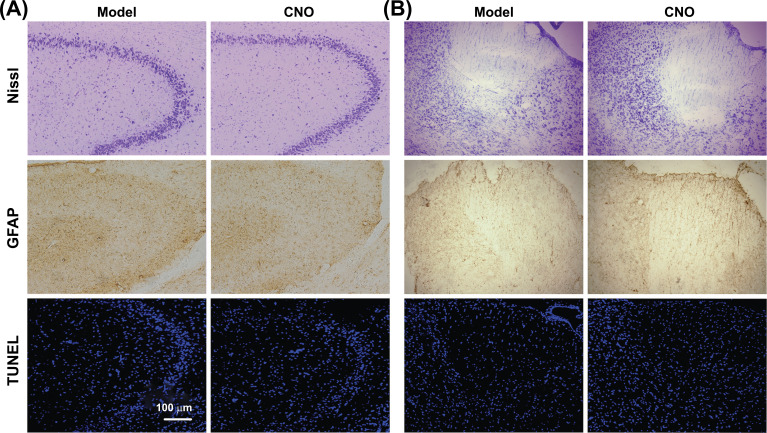
Chemogenetics produces non-neuropathology in rats. (**A**) Chemogenetic silencing of focal hippocampal seizures for local control. (**B**) Chemogenetic silencing of focal hippocampal seizures for remote control. Nissl staining revealed no detectable difference between the model group (left) and the CNO group (right). GFAP (Glial fibrillary acidic protein) staining of the model group (left) showed no significant difference with the CNO group (right). In TUNEL (TdT-mediated dUTP-biotin nick-end labeling) staining, no TUNEL-positive cells are discovered in the model group (left) and CNO group (right). The blue fluorescence in TUNEL staining comes from the Hoechst staining that is localized to nuclear DNA. Scale: 100 µm.

According to our Nissl, GFAP, and TUNEL staining results, no obvious neuronal necrosis, apoptosis, or glial cell proliferation was found in the hippocampus or ANT of rats in any group (Fig. **7**).

## 4 DISCUSSION

In the present study, we found that (1) both CNO and clozapine can locally inhibit 4-AP-induced focal hippocampal seizures chemogenetically with hM4Di-coding virus transfection on pyramidal cells in the seizure focus; (2) the inhibitory effect of CNO and clozapine also exists at a distal site from the seizure-onset zone (ANT), implying that ANT is a critical node in the epileptic network of temporal seizures; (3) the chemogenetic suppression of seizures by CNO and clozapine was time-dependent, and clozapine was faster and more effective than CNO in local seizure control; (4) the chemogenetic inhibition of seizures leads to a decrease in PSD in the theta and gamma bands; (5) neither the virus transfection process nor chemogenetics induce obvious neuropathology. These results provide direct evidence that chemogenetics exerted therapeutic effects on focal hippocampal seizures in rats, suggesting that chemogenetics may be a promising approach for the precise treatment of hippocampal seizures.

Hippocampal seizures are the most common type of epilepsy and are often not well-controlled by current treatments [39, 40]. Here, we first reported the use of a chemogenetic approach to attenuate hippocampal seizures by selectively inactivating pyramidal neurons in the seizure focus or an important node within the limbic seizure network. At present, the application of optogenetic technology has far outpaced chemogenetics, owing to the high temporal resolution of optogenetics, as well as its cell type and pathway-specific activation and silencing at the millisecond level [41]. While chemogenetics shares the benefits of precision treatment, and the advantages also lie elsewhere, making it more conducive for clinical transformation. First of all, unlike optogenetics, chemogenetics does not require the application of invasive and biocompatible devices to deliver light to transduced brain areas close to the seizure focus. Moreover, a relatively large area may be targeted because it is not limited by the absorption of light. For instance, CNO can be administered in multiple ways (such as intravenous, oral, and intraperitoneal injections), which makes chemogenetic technology adaptive and practical [9]. Additionally, the neuronal activation or silencing effect induced by the chemogenetic approach can last for several min to hours, ensuring a vital time for the treatment of chronic epilepsy after repeated administration [42]. Therefore, we believe that the use of chemogenetic inhibition of neuronal excitability to reduce seizures is a promising novel therapeutic modality for clinical use.

Anterior temporal lobectomy is the most common resection procedure for drug-resistant epilepsy. However, the postoperative seizure freedom rate is between 48% and 76% at one year [43, 44] and drops to 50% after 10 years [45]. One hypothesis underlying surgical failure is that an occult epileptogenic network, potentially composed of extratemporal regions, is already established preoperatively and spared during surgery, which develops into novel onset regions post-surgery and causes seizure recurrence [46-50].

Recently, chemogenetic techniques have been applied in models of focal epilepsy through the inhibition of pyramidal neurons in the hippocampus [14, 51], supporting the general applicability of chemogenetic approaches to control seizures despite diverse underlying pathophysiologies. Moreover, ANT has been suggested to be an extended hippocampal system due to its dense and reciprocal connections to the hippocampus and its involvement in cognitive function [52, 53]. Thus, while targeting a defined local focus may work for seizure control, we also examined the effect of targeting ANT, a node within the extended hippocampal system, also known as the limbic seizure network, to broaden the applicability of our method and to suggest the potential of disrupting epileptogenic network, which may help reduce the rate of recurrence according to previous studies. For the first time, we explored the possibilities of selectively inactivating pyramidal neurons with chemogenetic approaches at a distal site, ANT, in the attenuation of hippocampal seizures. Evidence from both clinical and preclinical studies has shown that deep brain stimulation (DBS) in the ANT suppresses epileptiform activity [54, 55]. Studies have suggested that DBS in the bilateral ANT decreases the SF in pilocarpine-induced epileptic rats [49]. Based on these considerations, we chose ANT as the target to control temporal lobe seizures using a chemogenetic approach with injected virus coding for inhibitory (hM4Di) DREADDs. In our study, we found that chemogenetics remotely inhibited focal hippocampal seizures by specifically inhibiting the excitability of pyramidal neurons in the ANT, indicating that the ANT plays an excitatory role in the thalamic-hippocampus circuit and emphasizes the importance of the ANT as a potential target in hippocampal seizures treatment. However, due to reports of cognitive difficulties after anterior thalamic stimulation for epilepsy control [31, 56], we also evaluated the effects of hM4Di expression on cognitive function in our rats using the Morris water maze test, a measure of memory function. Based on our results, no obvious influence was found on the learning and memory abilities of rats by viral infection in the hippocampus and ANT, suggesting that ANT is a potentially safe chemogenetic application site within the occult epileptogenic network for focal hippocampal seizure control.

The chemogenetic reduction of focal hippocampal seizures induced by 4-AP provides an interesting comparison of the effects of hippocampal and ANT suppression within the limbic circuit, highlighting the similarities and differences between the two approaches. In both cases, inhibition of pyramidal neurons in either node through CNO or clozapine application reduced seizure duration. However, a clear difference observed between the effects of local and remote control on hippocampal seizures was the reduction of SF in the clozapine group with local control. After the activation of the hM4Di receptor in the hippocampus, the cell membrane can be hyperpolarized [10], which significantly reduces the excitability of pyramidal neurons. Local control directly inhibits recurrent excitatory connections in the local hippocampal circuit of the seizure onset zone. In contrast, remote control reduced the excitability of the hippocampus to a limited degree through the projection of ANT in the limbic network while recruiting relatively more excitatory neurons in the epileptic initiation region than local control. This may explain why remote control is less effective than local control in suppressing the seizure duration. The reduction in hippocampal seizures by local and remote chemogenetic silencing in the limbic network adds further evidence regarding the potential mechanisms of action of network neuromodulation in epilepsy. Interestingly, changes in latency and SF were only observed in the clozapine group but not in the CNO group. Gomez *et al.* [18] reported that CNO can be converted into clozapine, which can quickly pass through the blood-brain barrier and subsequently act as a ligand to activate DREADDs in animal models. The much stronger latency prolonged and SF reduction effects of clozapine compared to CNO can potentially be explained by the pharmacological mechanism of CNO, which is metabolically converted to clozapine, which is the *in vivo* DREADD actuator. Recently, Zhang *et al.* [57] reported four DREADD-related cryogenic electron microscopy high-resolution structures, which may reveal key details of the recognition of DREADD chemogenetic actuators and the molecular basis for activation. In addition, the atypical antipsychotic drug, olanzapine, has been reported as a potent agonist of the human M4 muscarinic receptor-based DREADD, which may facilitate clinical translation of chemogenetics to treat central nervous system diseases [58]. All these findings should accelerate the structure-guided discovery of next-generation chemogenetic tools.

CNO is relatively metabolically inert in rodents, with variations in the order of a few percent [59], and can be reversibly metabolized to the parent drug, clozapine, in humans [60]. Gomez *et al.* [18] reported that CNO does not enter the brain after systemic injections, and clozapine, to which CNO rapidly converts *in vivo*, shows a high DREADDs affinity. However, clozapine, an activator of hM4Di, faces major logistical and regulatory obstacles. It is typically only prescribed for treatment-resistant patients because of its unfavorable side effects, including a higher risk of agranulocytosis and myocarditis in patients receiving a higher dose than in those at lower dosages [61-63]. According to Gomez *et al.* [18], while a low, subthreshold 0.1 mg/kg dose of clozapine significantly reduced locomotor activity in hM4Di-expressing rats but not in controls, a higher clozapine dosage (1 mg/kg) induced a decrease in locomotor activity, likely due to the sedative effect of clozapine at endogenous sites. They also showed that clozapine exhibited comparable and sufficient effects at both doses. Therefore, we adopted their subthreshold clozapine dose and provided further evidence that a 0.1 mg/kg dose of clozapine can effectively inhibit seizures induced by 4-AP in hM4Di-expressing rats but not in controls. The effectiveness of subthreshold clozapine in seizure treatment thus elevates the translational potential of chemogenetic technology owing to reduced side effects and more cost-efficient prescriptions. However, further studies are necessary to test and verify the safety and tolerance of clozapine in long-term anticytogenic efficacy in chronic models.

We observed a significant reduction in seizure duration and frequency within 10 min of clozapine administration and within 20 min of CNO administration in the local seizure control part, and this effect continued until the stable stage of the seizure model for more than 70 min. The model of the 4-AP-induced focal hippocampal seizure model was adopted in our experiment for the following reasons. First, a model that produces prolonged focal seizures soon after inducer administration is needed. This is because the ligand is normally taken into the brain within 5-10 min post injection [59], which introduces modest hyperpolarization and strong inhibition of axonal neurotransmitter release when it interacts with hM4Di [13, 64]. Second, recurrent electrographic focal seizures with hours of stable seizure duration and interictal period are needed, which is particularly suitable to test the effect of a new therapy expanding over a long period [14]. 4-AP-induced focal hippocampal seizures are an ideal model to examine the effects of chemogenetics on seizure control in ANT because they trigger temporal lobe histology and lasting alterations in excitability, allowing for precise experimental control over the timing of seizures.

The power of the theta and gamma bands was significantly reduced in focal hippocampal seizures in both local and remote sections in our study. There has been much research on the relationship between theta waves and seizure generation. Stypulkowski *et al.* [65] reported that inhibition of theta band power in DBS for large animals may be a potential mechanism for its epilepsy treatment effect. A previous study in a pilocarpine-induced rat epilepsy model reported that much of the increased preictal firing of neurons in the subiculum and CA1 correlated with preictal theta activity [66]. Moreover, in medically intractable hippocampal seizures, resection of theta band activity during the preictal period is essential for a good seizure outcome [67]. Another study of rapid focal cooling attenuated cortical seizures in a primate epilepsy model came to a similar conclusion [68]. These results suggest that inhibition of the theta band plays a key role in the attenuation of seizures. Wang and Liang *et al.* [69] found that low-frequency stimulation decreased the power of the delta band and increased the gamma band of hippocampal background EEG, but CNO did not interfere with the EEG (including the gamma band) during exploratory behavior in DREADDs (hM3Dq) mice [51]. Gamma rhythm is highly correlated with cognitive function [70, 71]. The decreased power of the gamma band induced by chemogenetics may contribute to improved learning and memory. Gamma oscillations in the hippocampus show clear regional and laminar patterns, and the CA3 region generates self-organized gamma oscillatory patterns [72-74]. Therefore, the decreased gamma band in the CA3 region in our study may stem from the chemogenetic suppression of neuronal network activities.

In addition, in both the local and remote control of hippocampal seizures, the percentage of LVF onset seizures decreased, while that of HYP onset seizures increased upon CNO or clozapine administration. A previous study suggested that the initiation of LVF and HYP onset seizures in the entorhinal cortex *in vitro* depends on the preponderant involvement of interneuronal and principal cell networks, respectively [4, 75]. They found that optogenetic activation of interneurons leads to LVF onset seizures, and activation of principal cells leads to HYP onset seizures. However, studies have shown that LVF and HYP seizures may mirror the activity of the distinct limbic structures [76]. Velasco *et al.* [37] reported that LVF seizures rest on the activity of the hippocampal and para-hippocampal networks, whereas HYP seizures originate from local circuits in the hippocampal formation. In addition, in kainic acid (KA)-treated rat models of hippocampal seizures, HYP onset seizures represent epileptogenic disturbances in hippocampal circuits, whereas LVF onset seizures may involve extrahippocampal areas [77]. In our study, local control selectively inhibited the excitability of pyramidal neurons in the hippocampus, which may have inhibited the spread of seizures, thereby limiting seizures within the hippocampus. This may explain why the percentage of LVF seizures decreased while that of HYP seizures increased. However, it was difficult to clarify which type of neuron was activated predominantly in the epileptogenic zone in the remote control of seizures through diverse and multi-projections. In addition, studies have reported that LVF initiation is more severe than HYP-onset seizures [78]; thus, the change in the seizure initiation mode from LVF to HYP may be a manifestation of reduced seizure intensity.

## CONCLUSION

Our study underscores the potential of locally and remotely inhibiting the excitability of pyramidal neurons using chemogenetics in the control of 4-AP-induced focal hippocampal seizures. We compared the effects of local and remote seizure control and found a faster response rate and shortened seizure duration in local control. We particularly shed light on epileptic network node disruption in seizure control, specifically on ANT, which plays an excitatory role in the limbic circuit, and the clinical translational value of clozapine in chemogenetic treatments of seizures.

## Data Availability

Not applicable.

## LIST OF ABBREVIATIONS

CNO: Clozapine‐N‐oxide
DBS: Deep Brain Stimulation
EEG: Electroencephalogram
HYP: Hypersynchronous
PBS: Phosphate Buffer Saline
PSD: Power Spectral Density
rAAV: Recombinant Adeno-associated Virus
SF: Seizure Frequency
TSD: Total Seizure Duration
